# Smartphone addiction among adolescents: associations with mental health, physical inactivity, daytime sleepiness, and consumption of ultra-processed foods

**DOI:** 10.1590/1984-0462/2025/43/2025019

**Published:** 2025-12-15

**Authors:** Lopes Laís Amaral, Bianca Borges Meireles, Luany Caxangá Carneiro, Carolina Amaral Oliveira Rodrigues, Hanna Beatriz Bacelar Tibães, Rosângela Ramos Veloso Silva, Lucineia de Pinho, Maria Fernanda Santos Figueiredo Brito

**Affiliations:** a Universidade Estadual de Montes Claros, Montes Claros, MG, Brazil.

**Keywords:** Addictive behavior, Smartphone, Smartphone addiction, Adolescent health, Adolescent, Comportamento aditivo, Smartphone, Adição ao smartphone, Saúde do adolescente, Adolescente

## Abstract

**Objective:**

To estimate the prevalence of smartphone addiction and identify associated factors among high school adolescents.

**Methods:**

A cross-sectional study, part of the first phase of the project “Longitudinal Study on Adolescent Behavior in Physical Activity and Health,” was conducted with 1,616 adolescents of both sexes from public schools in Montes Claros, Minas Gerais, Brazil. Sociodemographic data, lifestyle habits, health aspects, and future expectations were collected. Smartphone addiction was assessed using the Smartphone Addiction Inventory (SPAI) with a cutoff score of ≥9. Additionally, levels ofphysical activity, eating habits, daytime sleepiness, depressive and anxious symptoms, presence of pain, weight satisfaction, body mass index, waist-to-height ratio, and future perspectives were evaluated. Prevalence ratios (PR) with 95% confidence intervals (95%CI) were estimated using Poisson regression with robust variance in both crude and adjusted analyses (p<0.05).

**Results:**

The prevalence of smartphone addiction was 54.9%. Associated factors included female sex (PR 1.15; 95%CI 1.00–1.20), not practicing of physical activity (PR 1.10; 95%CI 1.00–1.22), consumption of salty industrialized/ultra-processed foods (PR 1.16; 95%CI 1.05–1.28) and fast foods (PR 1.16; 95%CI 1.04–1.28), presence of daytime sleepiness (PR 1.65; 95%CI 1.39–1.95), symptoms of depression (PR 1.42; 95%CI 1.22–1.65), and stress (PR 1.27; 95%CI 1.11–1.45).

**Conclusions:**

More than half of the adolescents demonstrated smartphone addiction, which was associated with female sex, unhealthy eating habits, and unfavorable emotional indicators. The findings highlight the importance of interventions that promote healthy behaviors and provide psychological support for this population.

## RESUMO

**Objetivo:**

Estimar a prevalência da dependência em smartphone e identificar os fatores associados em adolescentes do ensino médio.

**Métodos:**

Estudo transversal, integrante da primeira etapa do Projeto “Estudo Longitudinal sobre o Comportamento dos Adolescentes na Atividade Física e Saúde–, realizado com 1.616 adolescentes de ambos os sexos, de escolas públicas de Montes Claros, Minas Gerais, Brasil. Foram coletados dados sociodemográficos, de hábitos de vida, aspectos de saúde e expectativas futuras. A dependência em smartphone foi avaliada por meio do *Smartphone Addiction Inventory* (SPAI), com ponto de corte ≥9. Foram avaliados ainda o nível de atividade física, os hábitos alimentares, a sonolência, os sintomas depressivos e ansiosos, a presença de dores, a satisfação com o peso, o índice de massa corporal, a razão cintura-estatura e as perspectivas futuras. Razões de prevalência (RP) com intervalos de confiança de 95% (IC95%) foram estimadas por meio da regressão de Poisson com variância robusta, nas análises bruta e ajustada (p<0,05).

**Resultados:**

>A prevalência da dependência em smartphone foi de 54,9%. Os fatores associados foram: sexo feminino (RP 1,15; IC95% 1,00–1,20), não praticantes de atividade física (RP 1,10; IC95% 1,00–1,22), que consomem alimentos industrializados/ultraprocessados salgados (RP 1,16; IC95% 1,05–1,28) e *fast foods* (RP 1,16; IC95% 1,04–1,28), presença de sonolência diurna (RP 1,65; IC95% 1,39–1,95), sintomas de depressão (RP 1,42;IC95% 1,22–1,65) e estresse (RP 1,27; IC95% 1,11–1,45).

**Conclusões:**

Mais da metade dos adolescentes apresentaram a dependência em smartphone, a qual foi associada ao sexo feminino, hábitos alimentares não saudáveis e indicadores emocionais desfavoráveis. Os resultados ressaltam a importância de intervenções que promovam comportamentos saudáveis e apoio psicológico nessa população.

## INTRODUCTION

Smartphone use can lead to addiction, which is a growing concern worldwide. As the main device used to connect with technology, it is occupying more space in people’s daily lives. This smart, mobile, and compact device has a high capacity for data processing and offers services through the applications available in its operating system, such as internet access, social networks, news, games, and multimedia.^1,2,3^

Smartphones can increase productivity by accessing e-mails, facilitating social interaction through social networks, and expanding entertainment options, among other activities. Despite these benefits, excessive use of this technology can lead to dependence, a condition also described in the literature as smartphone “addiction.” However, there is widespread concern about the possible unfavorable effects of excessive use and how individuals may respond differently to prolonged exposure to the device.^4,5,6^

There is an intense debate about the use of digital technologies among adolescents, with concerns about social, cognitive, and affective risks.

This group is especially vulnerable to the changes brought about by technology and is exposed early to the use of smartphones and screens,^5^ which increases thetendency to develop addiction, driven by the constant search for new sensations.^2^

The prevalence of smartphone addiction varies between 4.3% and 70% among adolescents. This variation is strongly associated with the high technological penetration of this audience in certain countries.^7^ In addition, excessive use of the device by adolescents is related to a greater chance of developing complaints related to physical health, such as musculoskeletal pain^4^ and mental health, such as antisocial behavior, aggressiveness, sleep disorders, symptoms of anxiety and depression, attention, and learning disorders.^1,8^

Also noteworthy are compulsive behaviors, such as the constant and repeated checking of notifications; tolerance, observed by the duration and increase in the time of use; abstinence, manifested by restlessness or discomfort with the absence of the device; and functional impairment, caused by the interference of the cell phone in daily activities.^2^

Studies carried out in Brazil and Europe indicate that smartphone addiction harms lifestyle, negatively affecting mental and physical health. This condition interferes with eating habits and can contribute to increased adiposity, the development of eating disorders, and obesity among adolescents.^4,5,9,10^ However, Brazilian studies on smartphone addiction and its impacts among schoolaged adolescents are still scarce, highlighting the need for further investigation to prevent and/or mitigate its consequences.^7^

Therefore, the aim of this study was to estimate the prevalence of smartphone addiction and identify associated factors among high school adolescents.

## METHOD

This is an epidemiological, cross-sectional, and analytical study, part of the first stage of the ELCAS Project: “Longitudinal Study on Adolescent Behavior in Physical Activity and Health.” It was carried out with adolescents of both sexes from the state school system in the municipality of Montes Claros (MG), who were in their first year of high school in 2022 and 2023. This study was conducted and reported in accordance with the STROBE (Strengthening the Reporting of ObservationalStudies in Epidemiology) guidelines and was approved by the Research Ethics Committee of the State University of Montes Claros – UNIMONTES, under opinion no. 5.287.269/2022 and CAAE: 56165122.0.0000.5146.

The municipality had 43 schools in 2022, with a population of 3765 students enrolled in the first year of secondary school. For the sample calculation, a correction was made for a finite population, adopting a prevalence of 50%, a confidence level of 95%, an error of 3%, *deff=1*.5, and an addition of 10% for possible losses, estimating the need to survey at least 1373 adolescents.

The sample was selected using two-stage cluster probabilistic sampling, from urban schools. First, 20 schools were selected by probability proportional to size, and then, the classes were selected by simple random sampling. In rural schools, there was no selection process, as there were only two schools with first-grade classes. Students with regular enrolment were included, and those who were not present at the time of collection were excluded.

To assess smartphone addiction, we used the SPAI-BR^11^ version of the Smartphone Addiction Inventory (SPAI), translated, adapted, and validated for Brazilianindividuals. It consists of 26 dichotomous items (1=Yes; 0=No), which assess four factors: Compulsive Behavior, Functional Commitment, Abstinence, and Tolerance. The adolescents studied were classified into two groups according to the total score obtained on the instrument: non-problematic smartphone users (≤8 points) and problematic smartphone users (≥9 points).

The independent variables were sociodemographic: gender, age, skin color, single, social class, work, and study shift; lifestyle habits: level of physical activity andsatisfaction with leisure time; eating habits: consumption of industrialized/ultra-processed salty foods and consumption of fast foods; health aspects: daytime sleepiness, symptoms of depression, symptoms of anxiety, symptoms of stress, back or neck pain, satisfaction with weight, satisfaction with body image, excess weight, and waist-to-height ratio; and outlook: future expectations.

The sociodemographic variables were analyzed using the Brazilian Economic Classification Criterion (CCEB),^12^ an instrument that makes it possible to identify the socioeconomic profile of the participants, based on aspects such as education, occupation, and professional qualifications of those responsible. It also includes questions related to work during adolescence, type of employment, and the highest level of education achieved.

The level of physical activity was assessed using the International Physical Activity Questionnaire (IPAQ),^13^ a validated short version translated for Brazilian adolescents. The instrument is made up of eight questions, which allow us to estimate the weekly time spent on daily physical activity. Adolescents who practiced at least 60 minutes of regular physical activity a day were classified as “physically active” and those who did not reach a minimum of 60 minutes a day were classified as “not physically active.–

Satisfaction with leisure time was assessed using a question developed by the authors: “On a scale of 0 to 10, how do you rate your satisfaction with the non-compulsory activities you do?” The following cut-off points were established for interpretation: satisfied: 7 to 10 and dissatisfied: 0 to 6, categorized as “yes” and “no.”

Eating habits were assessed using a questionnaire applied in the National School Health Survey (PeNSE).^14^ Consumption of industrialized/ultra-processed salty foods and fast food was determined by an affirmative response to the consumption of at least one of the groups on seven days of the week. A response ≥5 days/week was considered regular consumption of the food in question and categorized as “yes” or “no.”

Daytime sleepiness was assessed using the Pediatric Daytime Sleepiness Scale (PDSS),^15^ translated and validated to assess Excessive Daytime Sleepiness (EDS) in Brazilian adolescents. This scale is made up of eight multiple-choice Likert-type questions, with the final sum of these scores ranging from 0 to 32 points. The cut-off point was adopted, which establishes 15 points as the limit for the PDSS,^16^ indicating the absence of daytime sleepiness at values below this mark. The participants were thus classified as “present” and “absent.”

To assess symptoms of depression, anxiety, and stress, the Depression, Anxiety and Stress Scale-21 (DASS-21)^17^ was used, a translated, adapted, and validated self-report instrument with 21 Likert-type questions. Each subscale assesses the three domains, with a score ranging from 0 to 21, which must be multiplied by two to obtain the final score. For the depression domain (items 3, 5, 10, 13, 16, 17, 21), the following scores were considered: 0 to 9 (normal), 10 to 13 (mild), 14 to 20 (moderate), 21 to 27 (severe), and above 28 (extremely severe). For anxiety, (items 2, 4, 7, 9, 15, 19,20), 0 to 7 (normal), 8 and 9 (mild), 10 to 14 (moderate), 15 to 19 (severe), and above 20 (extremely severe). And for stress, (items 1, 6, 8, 11, 12, 14, 18), 0 to 14 (normal), 15 to 18 (mild), 19 to 25 (moderate), 26 to 33 (severe), and above 34 (extremely severe). After calculating the final score, participants were grouped according to the presence (mild, moderate, severe, and extremely severe) or absence (normal) of symptoms related to each of the conditions assessed.

Back or neck pain was assessed using a question devised by the authors: “Are you experiencing back or neck pain at the moment?,” with “yes” and “no” answer options, and participants were categorized as “yes” or “no.”

Satisfaction with weight was assessed using a question created by the authors: “How do you feel about your body weight?,” with the answer options being “satisfied,” “neither satisfied nor dissatisfied” and “dissatisfied,” and the adolescents were categorized as “satisfied,” “neither satisfied nor dissatisfied” and “dissatisfied.”

To assess satisfaction with body image, the Silhouette Scale^18^ was used, consisting of 18 human silhouette figures, nine male and nine female, ranging from 1 (very thin) to 9 (very fat). The interviewee chooses two images, the first being the one that most resembles how they recognize themselves and the second the one that most resembles how they would like to be, answering the following questions: “Which silhouette most resembles you?” and “Which silhouette would you like to have?”

The score is calculated by the difference between the silhouette chosen as ideal and the current one, ranging from -8 to 8. If this variation was equal to zero, the adolescent was classified as satisfied with their appearance and, if it was different from zero, they were classified as dissatisfied. If the difference was positive, they were dissatisfied because they were overweight and, if it was negative, they were dissatisfied because they were thin. In the end, they were categorized as “satisfied” and “dissatisfied.”

To assess excess weight and the waist-to-height ratio, weight, height, and waist circumference were measured. The body mass index was calculated using the weight/height^2^ formula, stratified based on percentage values for the gender and age group, defined by the World Health Organization (WHO)^19^ and categorized as “yes” and “no.” The waist-to-height ratio^20^ was calculated by dividing waist circumference by height, considering a cut-off point of 0.5. Adolescents with values below 0.5 were considered “adequate” and those with values above 0.5 were considered “inadequate.”

The future expectations variable was assessed using a question prepared by the authors: “In relation to your future expectations, after finishing high school, do you…?,” with the answer options “intend to go to university,” “no” and “can’t answer” and categorized as “intend to go to university,” “no” and “can’t answer.”

After authorization from the 22^nd^ Regional Education Superintendence of Montes Claros-MG, data collection began in September 2022 and ended in December 2023. The research team was trained to standardize the approach to adolescents and calibrate the collection procedures. A pilot study was carried out in one of the selected schools to identify possible flaws in the proposed logistics and survey instruments and to estimate the average time taken to carry out the collection.

Visits were made to the selected schools to present the research project and schedule the collection. The students were informed, and the Informed Consent Form (ICF) was made available for parents/guardians to fill in. On the scheduled day, the ICF was collected, the Free and Informed Consent Form (FICF) was handed over, and the questionnaire was administered. Anthropometric measurements were taken in a private room.

IBM SPSS for Windows, version 22.0, was used for data analysis. Initially, descriptive statistics were calculated for all main variables. Bivariate analyses were conducted to identify associations with smartphone addiction. Variables with a p-value <0.20 were included in a multivariate model using Poisson regression with robust variance. In the final model, only variables with a statistically significant association (p < 0.05) were retained, and prevalence ratios (PRs) with 95% confidence intervals (CIs) were reported.

## RESULTS

The study included 1,616 adolescents, the majority of whom were female (50.4%), aged 15 years or younger (64.9%), nonwhite (73.9%), and single (95.1%). Regarding lifestyle and health habits, 65.4% were physically active, 68.2% reported satisfaction with leisure activities, and 72.0% presented with daytime sleepiness. In addition, a high proportion self-reported symptoms of depression (60.4%), anxiety (62.5%), and stress (53.7%). These and other characteristics are detailed in Table 1.

**Table 1:** Characterization of adolescents investigated in public schools in northern Minas Gerais. Montes Claros, Minas Gerais State, Brazil (n = 1616).

Variables	n^a^	%
Sociodemographic		
Sex (Female)	815	50.4
Age (Up to 15 years)	858	64.9
Skin color (Non-white)	1184	73.9
Single (Yes)	1505	95.1
Social class		
A	43	3.2
B	463	34.8
C	710	53.3
D/E	116	8.7
Employed (No)	1320	81.9
Shift in which attends school		
Full time	408	25.2
Morning	963	59.6
Afternoon	149	9.2
Evening	96	5.9
Lifestyle habits		
Level of physical activity (Physically active)	1004	65.4
Leisure satisfaction (Yes)	1083	68.2
Eating habits		
Consumption of salty industrialized/ultra-processed foodsb (No)	1107	68.5
Fast food consumption (Yes)	855	53.5
Health aspects		
Daytime sleepiness (Presence)	1131	72.0
Symptoms of depression (Yes)	907	60.4
Symptoms of anxiety (Yes)	951	62.5
Symptoms of stress (Yes)	803	53.7
Back or neck pain (No)	880	55.1
Weight satisfaction		
Satisfied	621	39.5
Neither satisfied nor dissatisfied	569	36.2
Dissatisfied	383	24.3
Body image satisfaction (Dissatisfied)	1101	71.1
Overweight (No)	634	76.3
Waist-to-height ratio (Adequate)	915	92.3
Perspectives		
Future expectations		
Intend to attend university	1028	64.8
Does not intend to attend university	157	9.9
Did not know how to answer	402	25.3
Smartphone addiction (Yes)	726	54.9

As for smartphone addiction, 50.4% of the students had this condition, showingasignificant prevalence among youngpeople. Table 2 presents thelogistic regression of factors associated with smartphone addiction among adolescents. After adjustments to the model, it was observed that female adolescents (PR 1.15; 95%CI 1.00–1.20), those whodo not engagein physical activity (PR 1.10;95%CI1.00–1.22), who consumesalty industrialized/ultra-processed foods (PR 1.16; 95%CI 1.05–1.28) and fast foods (PR 1.16; 95%CI 1.04–1.28), with the presence of daytime sleepiness (PR 1.65; 95%CI 1.39–1.95), symptoms of depression (PR 1.42; 95%CI 1.22–1.65), and stress (PR 1.27; 95%CI 1.11–1.45) were associated with higher prevalences of smartphone addiction.

**Table 2:** Smartphone addiction (n%) and associated factors among adolescents in public schools. Montes Claros, Minas Gerais, Brazil (n=1616).

Variables	Smartphone addiction		Crude PR (95%CI)	Adjusted PR (95%CI)
	No n (%)	Yes n (%)		
Sex (ref. = Male)				
Female	262 (44.0)	421 (58.0)	1.29 (1.16–1.42)*	1.15 (1.00–1.20)^†^
Level of physical activity (ref. = Physically active)				
Not physically active	155 (27.7)	268 (38.5)	1.23 (1.11–1.35)*	1.10 (1.00–1.22)^†^
Leisure satisfaction (ref. = Yes)				
No	160 (27.2)	246 (34.4)	1.15 (1.04–1.28)*	
Consumption of salted processed/ultra-processed foods (ref. = No)				
Yes	143 (24.0)	273 (37.6)	1.31 (1.19–1.44)*	1.16 (1.05–1.28)^†^
Fast food consumption (ref. = No)				
Yes	255 (43.1)	413 (57.4)	1.29 (1.17–1.43)*	1.16 (1.04–1.28)^†^
Daytime sleepiness (ref. = Absence)				
Presence	325 (55.9)	590 (83.6)	2.06 (1.76–2.42)*	1.65 (1.39–1.95)^†^
Symptoms of depression (ref. = No)				
Yes	249 (43.7)	512 (74.3)	1.89 (1.66–2.15)*	1.42 (1.22–1.65)^†^
Symptoms of anxiety (ref. = No)				
Yes	283 (48.9)	514 (73.6)	1.68 (1.48–1.90)*	
Symptoms of stress (ref. = No)				
Yes	209 (37.1)	462 (67.2)	1.77 (1.58–1.99)*	1.27 (1.11–1.45)^†^
Back or neck pain (ref. = No)				
Yes	227 (38.2)	376 (52.0)	1.28 (1.16–1.41)*	
Weight satisfaction (ref. = Satisfied)				
Neither satisfied nor dissatisfied	207 (35.4)	267 (37.7)	1.23 (1.08–1.39)*	
Dissatisfied	126 (21.5)	195 (27.5)	1.14 (1.01–1.28)	
Body image satisfaction (ref. = Satisfied)				
Dissatisfied	398 (69.5)	520 (73.7)	1.09 (0.98–1.23)	
Overweight (ref. = No)				
Yes	74 (23.4)	93 (24.5)	1.02 (0.87–1.20)	
Waist-to-height ratio (ref. = Adequate)				
Inadequate	29 (7.9)	36 (7.9)	0.98 (0.79–1.25)	
Future expectations (ref. = Intends to attend university)				
Does not intend to attend university	54 (9.2)	64 (8.9)	0.99 (0.83–1.18)	
Did not know how to answer	134 (22.9)	181 (25.2)	1.05 (0.94–1.18)	

PR: prevalence ratio; 95%CI: 95% confidence interval; Ref.: reference.

*p<0.20

^†^p<0.05.

## DISCUSSION

This study showed that more than half of the adolescents surveyed had smartphone addiction, an outcome that was associated with sociodemographic factors (gender); behavioral aspects (physical activity, consumption of industrialized/ultra-processed salty foods, consumption of fast foods, daytime sleepiness), and emotional aspects (depression and stress).

Internationally, the prevalence of smartphone addiction varies, with rates of 4.8% in Colombia,^21^ 33.3% in Jordan,^22^ and 95.8% in Cairo.^23^ In Brazil, this figure was 63.3% in a previous study of high school adolescents aged between 15 and 19.^24^ This variability can be attributed to differences in the methods used to investigate this condition, as well as to peculiarities related to different ethnicities, demographics, and cultures.

Being female was positively associated with smartphone addiction, in line with previous studies.^3,25^ Young women tend to use smartphones more to communicate, strengthen bonds and relationships, as well as to seek information and entertainment, which can favor the development of dysfunctional use of this tool.^25^

Smartphone addiction has been associated with physical inactivity. Research carried out with Chinese university students^9^ and Bangladeshi high school students^26^ found a negative association between physical activity levels and problematic smartphone use. This addiction can lead to excessive time spent on digitalactivities,^2^ reducing the daily time available for physical activity and increasing inertia.^27^ This condition can contribute to the development of overweight, obesity, and eating disorders.^5^

Smartphone addiction has been associated with increased consumption of industrialized/ultra-processed salty foods. A Spanish study found a positive relationship between increased consumption of these foods and increased exposure to screens.^5^ The use of smartphones contributes to this behavior, as the food industry uses theseplatforms to influence consumers. Adolescents are particularly vulnerable due to their susceptibility to digital marketing strategies.^5,10^

A higher consumption of fast foods was observed among adolescents with smartphone addiction. A previous study found that time spent using a smartphone is inversely proportional to vegetable and fruit consumption and directly proportional to consumption of sweets, carbohydrates, and fast foods. A positive relationship between screen time and unhealthy eating patterns has been observed among Korean adolescents.^28^

This behavior is related to the development of dietary restrictions, since internet users tend to adapt their eating patterns to the time spent on screens, skipping meals, and opting for fast foods.^9^ This condition contributes to a reduced quality of life.^29^

Smartphone addiction has been associated with daytime sleepiness. Nighttime use of more than two hours impairs sleep in adolescents, making it non-restorative. Excessive exposure to blue light before bedtime reduces melatonin secretion and alters the circadian cycle, causing sleep disturbances. This generates psychophysiological arousal, reducing relaxation, and delaying sleep induction.^2,30^ Excessive daytime sleepiness affects cognition and school performance.^30^

A significant association was found between symptoms of depression and smartphone addiction, which was also found in Chinese medical students^4^ and Korean high school students.^3^ Excessive smartphone use affects neuropsychological development^22^ and is related to the problematization of social relationships by adolescents, leading them to choose loneliness and experience depressive symptoms.^8^ The opposite is also true, with depressed people tending to use their smartphone excessively as a means of relieving negative feelings.^5,23^

Smartphone addiction has been associated with stress symptoms. The association between the severity of smartphone addiction and stress has been observed among university students.^31^ A Korean study showed that the greater the stress, the greater its influence on smartphone addiction.^3^ Faced with stressful situations, adolescents use escape mechanisms, choosing to immerse themselves in the virtual world in order to ignore and avoid facing real problems.^3^ On the other hand, a Brazilian study found that the majority of participants with addiction were unable to cope with stress.^27^ The low levels of self-control caused by the abusive use of this device may explain the higher levels of stress in this population.^2^

Limitations of the study include the cross-sectional design, which, although useful for determining the prevalence of certain conditions, presents difficulties in assessing the temporal sequence of the independent variables and the outcome, thus making it impossible to determine a causal relationship. Also noteworthy is reverse causality, which suggests difficulties in determining the primary cause and which external factors may influence smartphone addiction. Other limitations include the investigation of adolescents from a single grade and the use of self-reported data to assess behavioral and emotional aspects, although protected by the use of a validated instrument. Thus, adolescents may tend to report more socially acceptable responses, to the detriment of reality, which they may consider problematic. However, the results are based on a representative sample, which increases their robustness.

At the time of data collection, there was no specific instrument to assess the criteria for smartphone addiction in adolescents. This may have influenced the accuracy of the results,since smartphone usage patterns may vary according to the age group. However, the instrument used is validated and widely recognized in the literature, allowing for comparison of results with other studies in the field.

In conclusion, this study found a high prevalence of smartphone addiction among high school adolescents, with a higher prevalence among female adolescents. The data suggest associations between smartphone addiction and not being physically active, consumption of industrialized/ultra-processed salty foods, consumption of fast foods, daytime sleepiness, symptoms of depression, and symptoms of stress. This knowledge can improve our understanding of this condition and support actions to promote health and prevent, monitor, and control this condition, considering the associated factors.

## Data Availability

The database that originated the article is available with the corresponding author.
