# ERRATUM: Smartphone addiction among adolescents: associations with mental health, physical inactivity, daytime sleepiness, and consumption of ultra-processed foods

**DOI:** 10.1590/1984-0462/2026/44/2025019-ER

**Published:** 2026-06-19

In the manuscript: Amaral LL, Meireles BB, Carneiro LC, Rodrigues CAO, Tibães HBB, Silva RRV, et al. Smartphone addiction among adolescents: associations with mental health, physical inactivity, daytime sleepiness, and consumption of ultra-processed foods. Rev Paul Pediatr. 2025;43:e2025019. https://doi.org/10.1590/1984-0462/2025/43/2025019

**On page 4, 1st column, 4th paragraph**

**Where it reads:**

Variables with a p-value <0.20 were included in a multivariate model using Poisson regression with robust variance.

**It should read:**

Variables with a p-value <0.20 were included in a multiple model using Poisson regression with robust variance.

**On page 4, 1st column, 6th paragraph**

**Where it reads:**

As for smartphone addiction, 50.4% of the students had this condition, showingasignificant prevalence among youngpeople.

**It should read:**

As for smartphone addiction, 54.9% of the students had this condition, showingasignificant prevalence among youngpeople.

